# Design and Development of a Non-invasive Opto-Electronic Sensor for Blood Glucose Monitoring Using a Visible Light Source

**DOI:** 10.7759/cureus.60745

**Published:** 2024-05-21

**Authors:** Iftekar Alam, Anjaneyulu Dunde, Kartheek R Balapala, Moumita Gangopadhyay, Saikat Dewanjee, Moutima Mukherjee

**Affiliations:** 1 Department of Medical Physics, Adamas University, Kolkata, IND; 2 Department of Internal Medicine, Baptist Medical Center South, Montgomery, USA; 3 Department of Physiology, Michael Chilufya Sata School of Medicine, The Copperbelt University, Kitwe, ZMB; 4 Department of Bio-Technology, Adamas University, Kolkata, IND; 5 Department of Pharmaceutical Technology, Jadavpur University, Kolkata, IND

**Keywords:** patient compliance in diabetes care, commercially viable medical devices, optical glucose sensing, glucose concentration detection, physiological variability, infrared spectroscopy, blood glucose measurement, laser light refraction, diabetes management, non-invasive glucose monitoring

## Abstract

Background

The management of diabetes is critically dependent on the continuous monitoring of blood glucose levels. Contemporary approaches primarily utilize invasive methods, which often prove to be uncomfortable and can deter patient adherence. There is a pressing need for the development of novel strategies that improve patient compliance and simplify the process of glucose monitoring.

Aim and objectives

The primary objective of this research is to develop a non-invasive blood glucose monitoring system (NIBGMS) that offers a convenient alternative to conventional invasive methods. This study aims to demonstrate the feasibility and accuracy of using visible laser light at a wavelength of 650 nm for glucose monitoring and to address physiological and technical challenges associated with in vivo measurements.

Methods

Our approach involved the design of a device that exploits the quantitative relationship between glucose concentration and the refraction phenomena of laser light. The system was initially calibrated and tested using glucose solutions across a range of concentrations (25-500 mg/dL). To get around the problems that come up when people's skin and bodies are different, we combined an infrared (IR) transmitter (800 nm) and receiver that checks for changes in voltage, which are indicative of glucose levels.

Results

The prototype device was compared with a commercially available blood glucose monitor (Accu-Chek active machine; Roche Diabetes Care, Inc., Mumbai, India). The results demonstrated an average linearity of 95.7% relative to the Accu-Chek machine, indicating a high level of accuracy in the non-invasive measurement of glucose levels.

Conclusions

The findings suggest that our NIBGMS holds significant promise for clinical application. It reduces the discomfort associated with blood sampling and provides reliable measurements that are comparable to those of existing invasive methods. The successful development of this device paves the way for further commercial translation and could significantly improve the quality of life for individuals with diabetes, by facilitating easier and more frequent monitoring.

## Introduction

Diabetes is a prevalent metabolic syndrome attributed to an elevated blood glucose level [1]. It is considered a major cause of death and a decline in life expectancy. Diabetes is a silent killer that can promote a variety of pathological conditions, including microvascular complications, peripheral and coronary artery diseases, and stroke. Despite several measures being taken to fight against diabetes, the prevalence remains alarmingly uncontrolled. According to the International Diabetes Federation, the number of diabetes adults (20-79 years old) was 537 million in 2021, and it is anticipated to reach 643 million by 2030 [2]. The International Diabetes Federation also prognoses that about 783 million people, i.e., one in eight adults, will suffer from diabetes by 2045, a serious 46% rise [2]. The primary cause of diabetes-associated mortality has been proposed to be hyperglycemia-induced oxidative stress, which endorses several pathological processes [3-6]. Although there is presently no cure for diabetes, restoring blood glucose levels to near-normal levels is the most important part of diabetes treatment to avoid diabetes-associated complications and increase the lifespan of patients. The daily monitoring of blood glucose levels is recommended for diabetic patients receiving insulin therapy [7,8]. Although patients using oral antidiabetic medications do not require regular monitoring of blood glucose, patients taking specific drug classes (sulfonylurea) that have the potential to cause hypoglycemia require regular monitoring of blood glucose levels [8,9]. Overall, all diabetic or prediabetic patients require blood glucose monitoring at specific intervals based on the severity of hyperglycemia, diabetes type, and medication.

Among the different types of blood glucose monitoring, capillary blood glucose and venous blood sample analysis are two common invasive techniques for blood glucose measurements [10,11]. In the capillary blood glucose test, the glucose levels can be measured at home from a small blood sample obtained from the middle finger using glucometers, but the strips are expensive, and the accuracy and reliability of the results are dependent on several factors, including machine age [10-12]. Though later gives more accurate results, the analysis can only be done in clinical laboratories. Moreover, both are repetitively painful processes and expensive for regular blood glucose monitoring [13]. Thus, the development of a dependable, non-invasive blood glucose monitoring system (NIBGMS) is a need-based attempt at regular monitoring of blood glucose, considering the global threat of diabetes prevalence. The detection of glucose levels through the skin using different spectroscopic analyses has been explored [14-19]. However, the drawbacks of these methods, such as poor linearity and sensitivity, a low signal-to-noise ratio, sensitivity to changes in the environment, imprecision, and dependence on skin composition, limited their potential to serve their purpose. Other approaches, such as reverse iontophoresis, bioimpedance spectroscopy, ultrasound, and electromagnetic sensing, have also been explored; however, they are subjective to ambient changes, skin thickness, and physiological time lags in blood glucose [20-23].

Here, we aimed to develop a NIBGMS employing 650 nm visible laser light, which shows superior penetrability, signal-to-noise ratio, and accuracy, compared to near-infrared (NIR) spectroscopy at 950 nm. The quantitative relationship between the concentration of the glucose solution and the change in refraction phenomena was considered in designing the device. Finally, the device was calibrated considering the quality and physiological properties of the skin, by monitoring the voltage drop involving an infrared transmitter (800 nm) and an infrared receiver.

## Materials and methods

**Figure 1:**
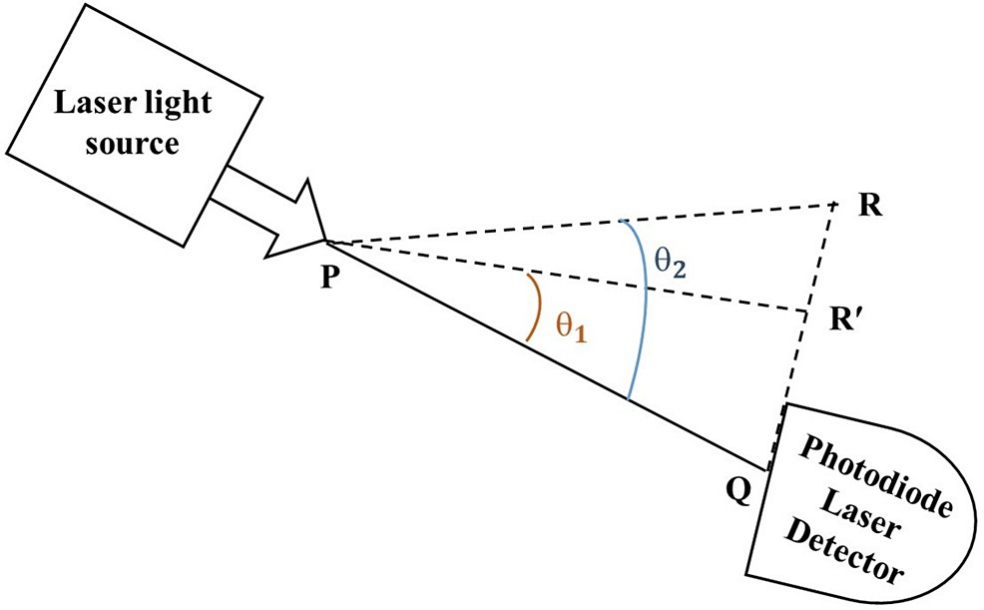
The refractive angle decreases as glucose concentration rises PQ has a fixed distance of 75 mm PR, PR', and PQ: light ray distance; θ1 and θ2: refractive angles

Glucose molecules can modify the angle of refraction of light in proportion to their concentration and the medium's overall refractive index. The refraction-based estimation is based on Snell's law, and the magnitude of each parameter correlates with the glucose concentration in the aqueous solution. The refractive index of each medium influences the bending of light as it transitions between them, following Snell's law, also known as the law of refraction. In glucose monitoring, this principle is applied to measure glucose concentrations based on changes in light refraction through an aqueous glucose solution. As glucose levels vary, they alter the solution's refractive index, consequently adjusting the light's refraction angle. By observing these changes in the angle of refraction, one can accurately determine the glucose concentration, utilizing the inverse relationship outlined in Snell's law. As the glucose concentration increases, the light ray (PR) in Figure 1 tends to tilt toward the normal PQ and the refractive angle decreases; hence, the photodiode receives a greater number of photons.

**Figure 2:**
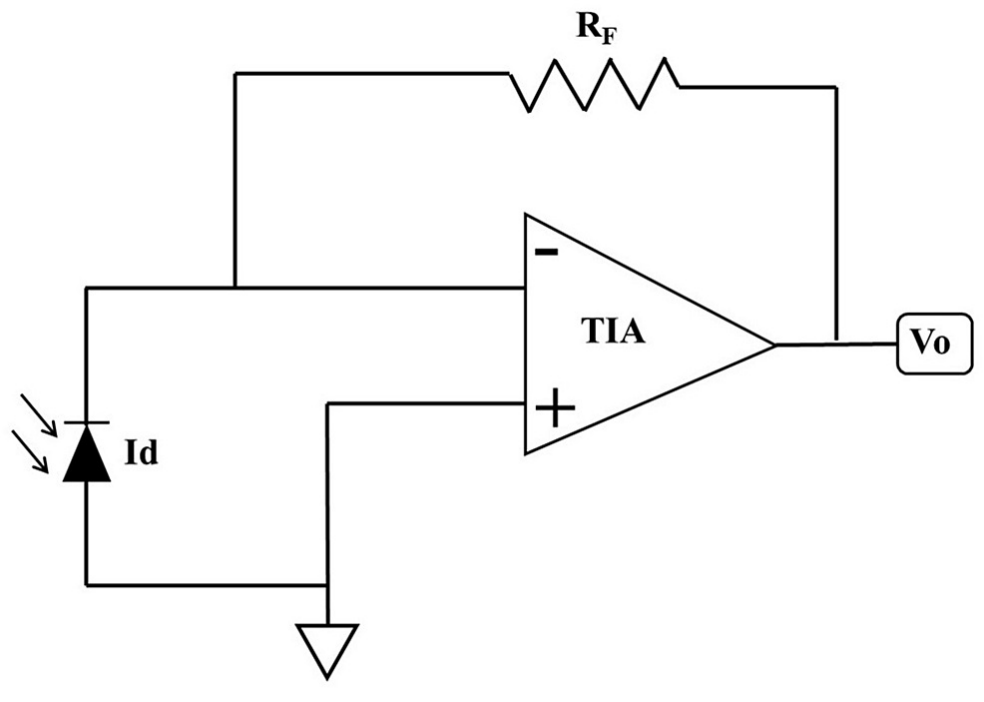
Trans-impedance amplifier to convert photodiode current to voltage TIA: trans-impedance amplifier; Vo: voltage output; Id: photodiode current; RF: feedback resistor

Using a trans-impedance amplifier (TIA), the photodiode current is converted into a voltage output. The TIA circuit detects incoming radiant radiation and then outputs an equivalent voltage. As shown in Figure 2, the TIA equivalent circuit can be utilized to calculate the output voltage (Vo).

Vo = Id × RF, where Vo represents the voltage output, Id denotes the photodiode current, and RF stands for the feedback resistor used as the gain of the TIA.

Challenges during in-vivo experiment

The absorption of visible laser light by human skin is one of the most challenging aspects of in-vivo experimental studies for noninvasive blood glucose monitoring. Human skin is a highly absorbent substance, and depending on the thickness of the subject's skin, the rate of absorption may differ between individuals. Depending on factors such as skin pigmentation, amount of wetness, and the presence of other substances on the skin's surface, the skin's absorption capacity might vary. Due to these changes, it may be difficult to obtain consistent and reliable blood glucose measurements, which may introduce doubt into the test results. To account for the skin's propensity to absorb light, it may be necessary to employ calibration procedures or correction factors. In addition, signal processing and filtering can be utilized to improve signal-to-noise ratio and measurement precision. To account for changes in skin quality and ensure the system's functionality on all skin types, extensive testing and validation with a large number of individuals are required.

Calibration of intensity for skin thickness

A non-invasive technique for measuring epidermal thickness utilizes the intensity of transmitted near-IR radiation, as outlined in the reference [24]. This method employs a simple model wherein light is transmitted through the epidermal layers, and the reflected light from the surface is measured. The experimental setup includes an adjustment system to accommodate variations in skin thickness among different patients. Specifically, the system comprises an IR transmitter (operating at 800 nm and 1.4 V) and an IR receiver. The chosen voltage of 1.4 V ensures optimal power output and device safety, as it balances effective IR emission for tissue penetration and minimizes risk to the skin. The IR light is directed onto the patient's finger, and the intensity of the light diminishes based on factors such as the skin's thickness. The IR receiver then captures the light that is reflected back from the finger, providing data crucial for determining epidermal thickness. The thicknesses of the skin are indirectly measured by observing the voltage drop, as shown in Table 1.

**Table 1:** Calibration of input laser intensity for different skin thicknesses V: voltage; mW: milli Watt; IR: infrared

Skin type	Incident IR voltage (V)	Receiver voltage (V)	Voltage drop (V)	Input laser intensity adjustment (mW)
1	1.4	0.45	<=0.95	5
2	1.4	0.4	0.96-1.0	6
3	1.4	0.35	1.01-1.05	7
4	1.4	0.3	1.06-1.1	8
5	1.4	0.25	1.1-1.15	9
6	1.4	0.2	1.16-1.2	10

The Arduino plays a critical role in our experimental setup by transmitting voltage difference data to a command board. This command board is specifically programmed to analyze the transmitted voltage differences and compare them against predefined voltage drops associated with various patients (Table 1). This comparison is crucial for determining the optimal laser light intensity required for each patient. By dynamically adjusting the laser light intensity, our device compensates for discrepancies in skin thickness among different patients. This adaptive approach ensures that the measurements remain consistent and accurate across a diverse patient population. Employing this method to regulate the intensity significantly enhances the precision of our NIBGMS, which utilizes visible laser light. By effectively minimizing the influence of variable skin thickness, we are able to improve the overall accuracy of glucose level readings.

NIBGM prototype circuit

**Figure 3:**
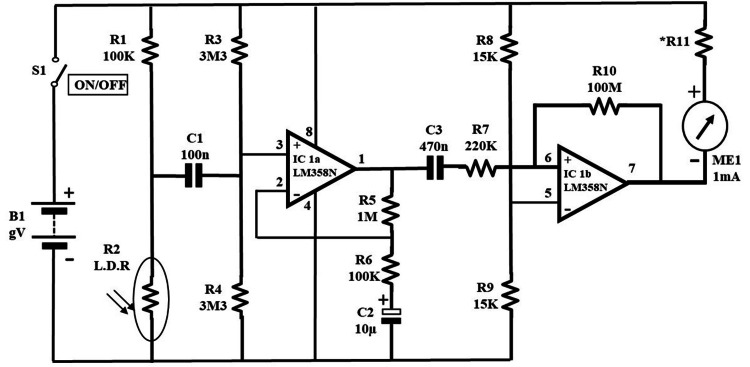
Circuit diagram of NIBGM prototype NIBGM: non-invasive blood glucose monitor; R1-R11: resistors; C: capacitor;  LDR: light-dependent resistor; Op-Amp: operational amplifier; mA: milliampere; IC1b: inverting operational amplifier; ME1: metallic enclosure 1; K: kilo ohms; M: mega ohms

Figure 3 depicts the NIBGM prototype based on visible laser light. When a finger is put into the finger hole, the light-dependent resistor (LDR) measures the changes in light intensity caused by fluctuations in blood glucose levels and skin characteristics. Variations in light intensity are translated into voltage and amplified using a two-stage amplifier circuit comprising a non-inverting operational amplifier (Op-Amp) IC1a and an inverting Op-Amp IC1b. The gain of roughly 800, calculated by resistors R5, R7, and R10, provides the amplification. The amplified voltage signal carrying information about blood glucose levels is produced by the output of the second Op-Amp (pin 7 of IC1b). This signal can be further processed or displayed to estimate glucose levels. To avoid stray light from reaching the LDR, a little button-style LDR is suggested over a larger LDR like ORP12.

NIBGM hardware implementation

**Figure 4:**
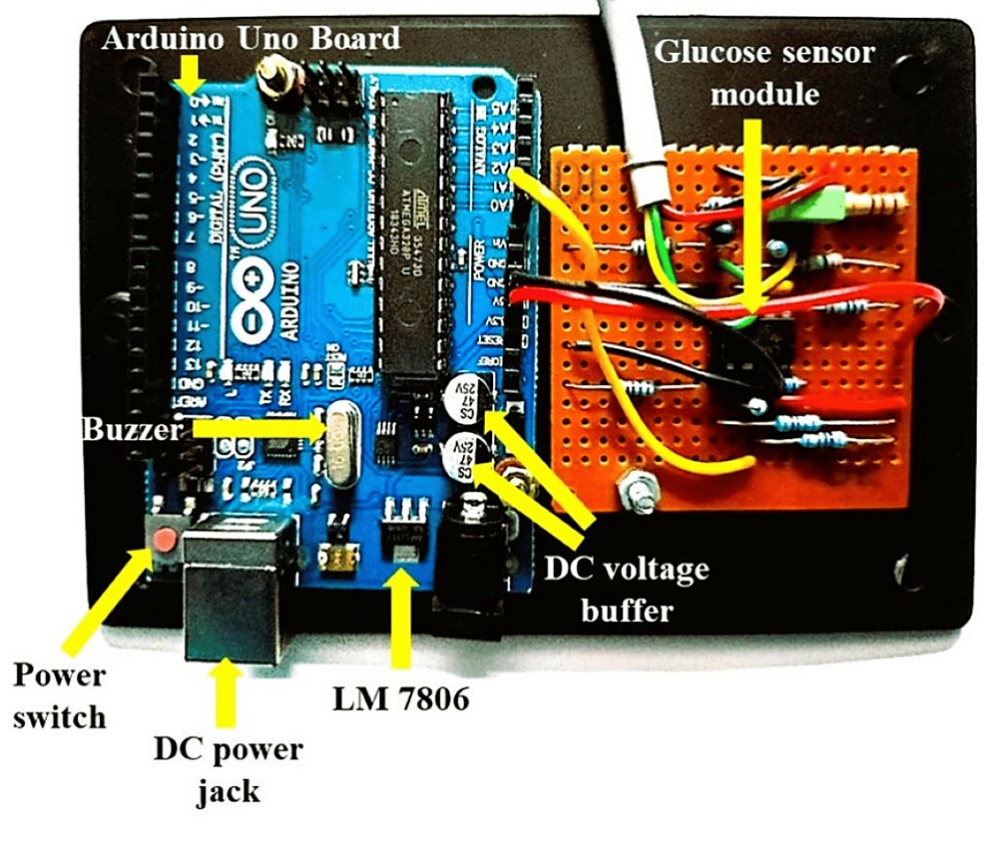
NIBGM prototype setup for real-time monitoring of glucose DC: direct current; LM: linear monolithic; NIBGM: non-invasive blood glucose monitor

The hardware implementation of the NIBGM prototype is illustrated in Figure 4.

The glucose sensor module, Arduino Uno board, power supply, and voltage buffer are the key components of the setup. Arduino Uno board is a versatile microcontroller based on the ATmega328P, widely used in electronics projects for its ease of use and extensive support. It features 14 digital input/output pins, six analog inputs, USB connectivity, and can be programmed via the Arduino IDE a language similar to C++, making it ideal for prototyping applications. The KY-008 visible red laser source, the OPT101 integrated photodiode with an on-chip TIA, and the finger hole compose the glucose sensor module. The KY-008 laser source emits visible red light with a wavelength of 650 nm and an adjustable intensity range from 5 to 10 mW. The integrated photodiode OPT101 detects laser light, amplifies the current, and transforms it into a voltage output. The finger hole is designed to prevent external ambient light interference from interfering with accurate measurements. The Arduino Uno board has an ATmega328P microprocessor, which is responsible for determining blood glucose levels based on photodiode voltage output. The microcontroller executes the necessary computations and shows the results on the Arduino Project software, allowing for real-time monitoring and assessment of glucose levels. A 12VDC power adaptor, a DC power jack, a power switch, and an LM7805 voltage regulator comprise the power supply. The power adaptor provides the circuit with the necessary power and the voltage regulator guarantees that the components receive a stable and controlled voltage supply. Two LM358 Op-Amps are utilized in the DC voltage buffer to remove any DC offsets from the voltage output. This helps to avoid any undesired voltage fluctuations and ensures a more precise glucose concentration reading.

Study subjects

The non-invasive blood glucose monitoring device was tested on a cohort of 198 volunteers, comprising both men and women, aged 20 to 60 years. These participants underwent testing with the new device both before and two hours after consuming breakfast. The study received ethical approval from Adams University, Kolkata, India, under the authorization number AU/IEC/BT/2023-5.

## Results

In-vitro measurements

**Figure 5:**
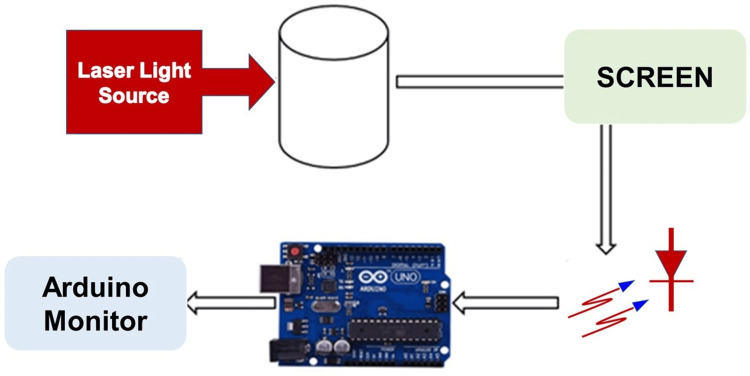
Experimental setup for in-vitro studies This figure illustrates the experimental setup used to measure glucose concentrations. The photodiode is positioned beneath the sample holder to capture transmitted light through varying concentrations of glucose solutions. As glucose concentration changes, the photodiode detects variations in light intensity, converting these into a corresponding voltage output. The voltage signals are then recorded and monitored in real-time using Arduino software on a connected PC

Various quantities of glucose-D aqueous solutions in distilled water were used in the in-vitro investigations. By passing red laser light through a glucose solution, the parameters of laser refraction were measured. On the screen, the laser's circular spot and refraction properties were measured. The photodiode was placed underneath the sample holder to record the voltage output as it changed in response to glucose concentration variations. The photodiode detected the transmitted light and translated it into an equivalent voltage output, which was monitored using Arduino software on a PC (Figure 5).

**Figure 6:**
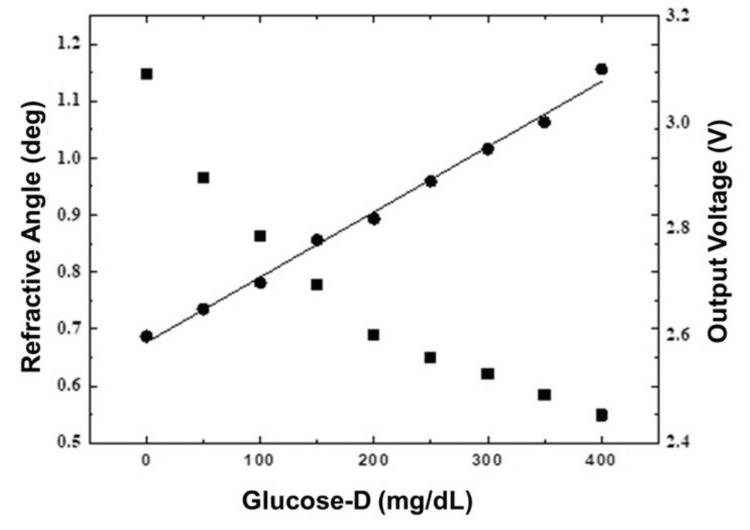
The graph displays changes in refractive index (squares) and output voltage (circles) against glucose-D concentration It highlights a direct correlation between glucose concentration and refractive angle, and the output voltage data follows a linear trend mg: milligram; dL: deciliter; glucose-D: dextro glucose

As shown in Figure 6, Snell's law predicts that as the concentration of glucose-D rises, the refractive angle will decrease, increasing the refractive index. Furthermore, when the concentration of the glucose solution grows, so does the Arduino voltage output. The following equation can be used to describe the data: Y = 47.186X + 27.246.

**Figure 7:**
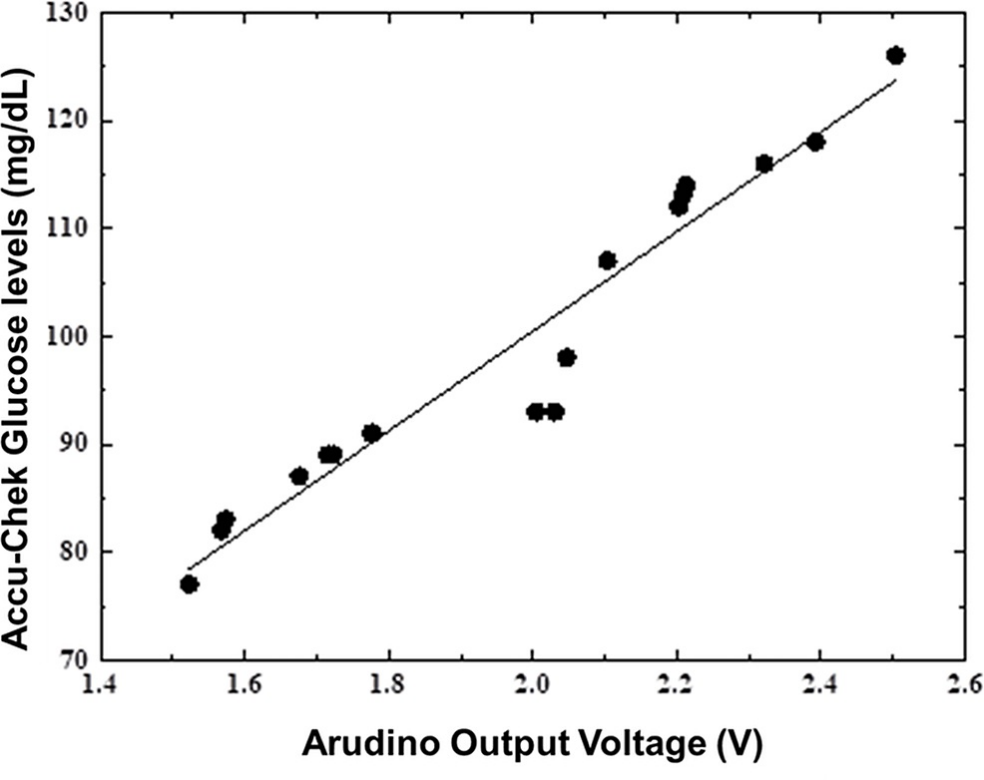
Calibration between the Arduino output voltage and Accu-Chek glucose levels The line is fit to a straight line mg: milligram; dL: deciliter

Later, as shown in Figure 7, the output voltage was correlated to Accu-Chek glucose output (Roche Diabetes Care, Inc., Mumbai, India). This confirms the consistency of the suggested approach of non-invasive blood glucose monitoring using the red laser.

In-vivo measurements

**Figure 8:**
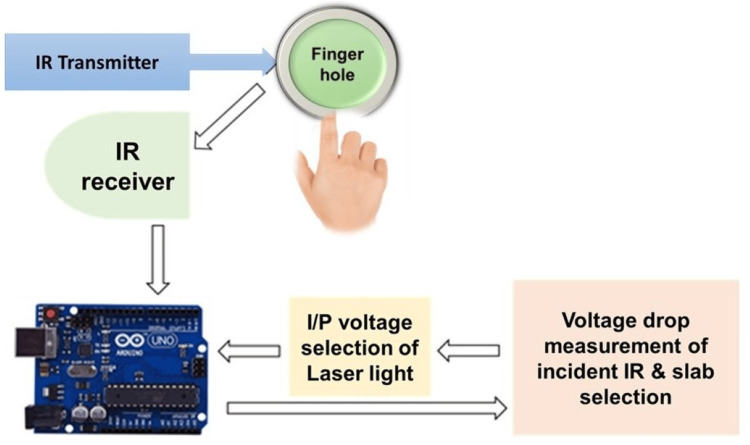
Schematic setup of the final device used to acquire the glucose levels of patients IP: input voltage; IR: infrared

The experimental setup depicted in Figure 8 was used to take in-vivo measurements on 198 volunteers, both male and female, aged 20-60 years, before and two hours after breakfast.

**Figure 9:**
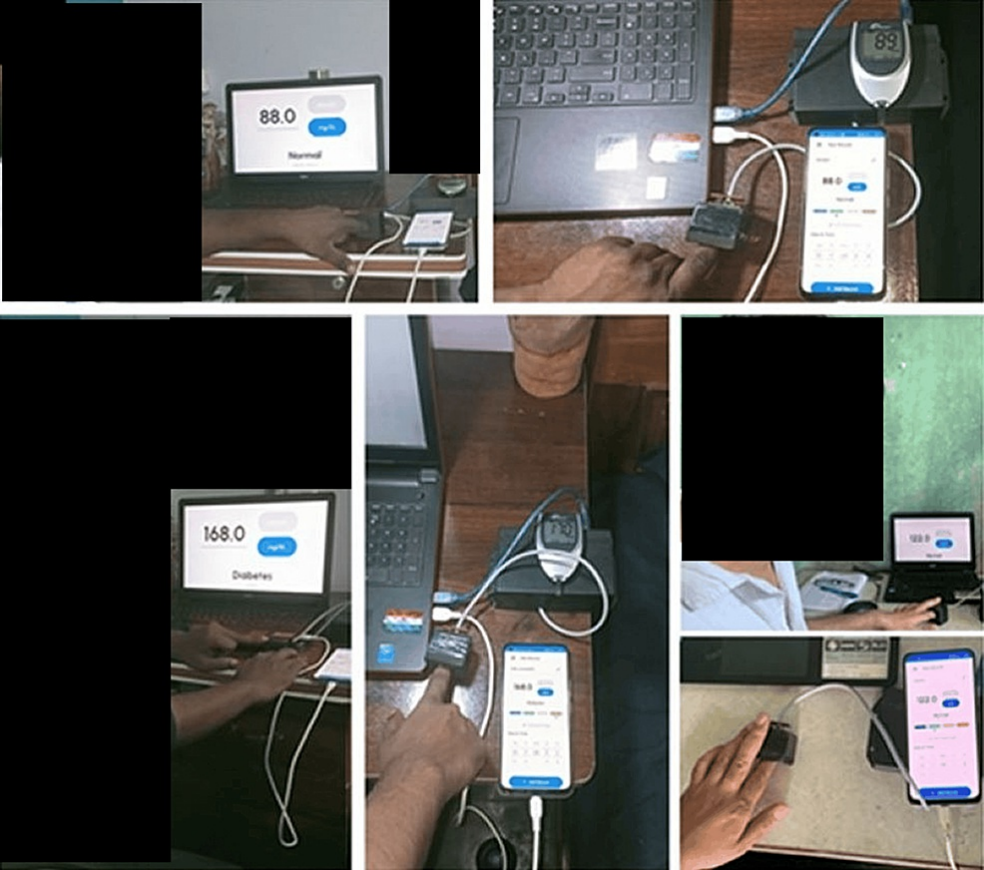
Comparison of blood glucose levels of our designed device with a commercially available glucometer and real-time monitoring of glucose levels of a patient using a smartphone or a laptop

The Arduino board automatically adjusted the intensity of the incoming laser light based on the thickness of each subject's finger skin. As shown in Figure 9, with the designed prototype, the measurement of the glucose levels of a patient can be monitored either on the smartphone or on the laptop, which is very handy, and the values are comparable to those of commercially available glucometers such as the Accu-Chek active machine.

**Figure 10:**
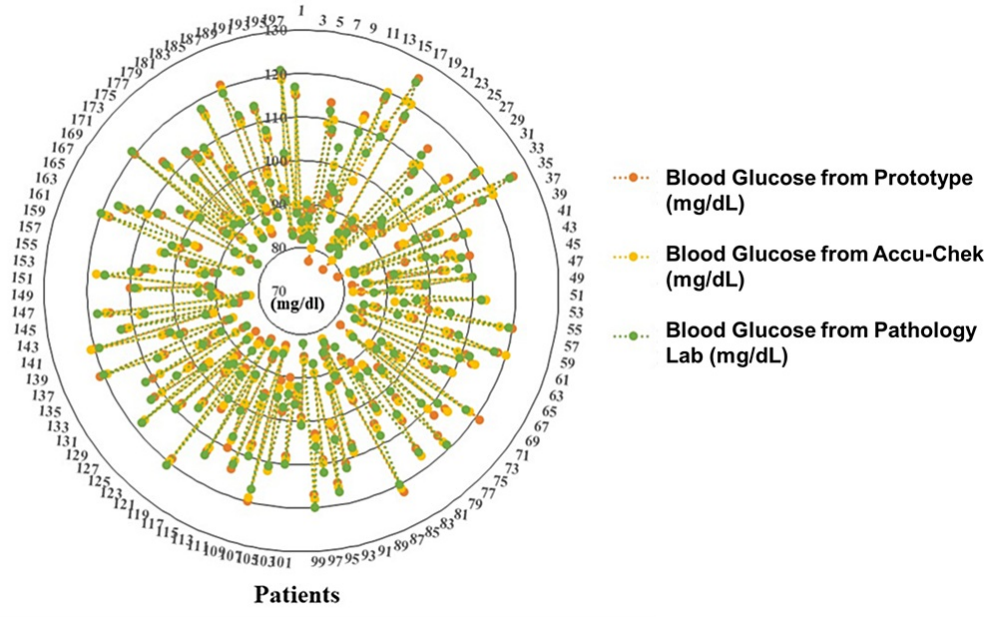
Comparison between measurements of blood glucose levels measured non-invasively with the proposed prototype (orange dots), invasively by active Accu-Chek machine (yellow), and from pathology lab (green) The vertical scale is the blood glucose level in mg/dL for the 197 patients/samples

A small demonstration of the developed non-invasive optoelectronic sensor has been displayed in Figure 9. In addition, the data obtained from the NIBGM device was compared with pathological glucose test results. Figure 10 shows a comparison of the NIBGM blood glucose values to those of an invasive commercial Accu-Chek blood glucose monitor and pathology lab results with error computation. It has an error of less than 4% with commercial invasive devices and an inaccuracy of less than 5% with pathology lab results.

## Discussion

The discussion in the paper provides a detailed analysis of the technology used in the development of a non-invasive blood glucose monitoring system, emphasizing the choice of specific wavelengths in comparison to those used in previous research. Prior studies have identified that certain NIR wavelengths, particularly those within the range of 1500-1850 nm and 2050-2392 nm, are optimal for blood glucose monitoring because they minimize water absorption and enhance signal strength [25]. This is important because less water absorption leads to clearer glucose signal detection, which is crucial for accurate measurements.

However, many researchers have opted to use shorter NIR wavelengths, between 800 and 1350 nm. This decision is based on the need for deeper penetration into the skin, which is essential for capturing detailed spectral data from the skin's lower layers, despite the challenge of higher water absorption at these wavelengths [26,27]. The ability of these shorter wavelengths to penetrate deeper makes them valuable, although they introduce some level of measurement complexity due to the interference from water in the body. In addition to NIR technologies, the paper notes that visible-to-infrared (Vis-IR) biosensors have also been utilized in previous glucose monitoring research [28]. These biosensors combine the capabilities of visible light and IR technology, providing a versatile approach to detecting glucose levels beneath the skin's surface.

In developing their device, the authors selected an 850 nm IR LED and a visible spectrum red laser. The fact that LEDs are readily available on the market and affordable made these decisions possible. While these wavelengths do not completely avoid water absorption, they offer a practical compromise between effective skin penetration and manageable spectral interference. This makes the device both functional and economically viable for regular use by patients.

The device demonstrated an impressive 96% accuracy rate in trials, highlighting its effectiveness compared to traditional and other non-invasive methods. This high level of accuracy was consistent across different age groups and testing environments, showcasing the device's robustness. The researchers conducted extensive tests, both in laboratory settings and in real-world conditions, to ensure that the device could reliably measure glucose levels in diverse scenarios.

The Internet of Things (IoT), particularly known as the Internet of Surgical Things (IoST), has significantly evolved to enhance the technological landscape of surgery, integrating a network of internet-connected devices capable of sensing, actuating, and data processing. Primary applications such as telesurgery and surgical telementoring utilize these connected devices for conducting or mentoring surgeries remotely. This system facilitates the transmission of audiovisual cues and control commands across great distances, enabling operations by remote surgeons or guidance by experienced mentors [29]. Additionally, the scope of IoT extends to image-guided surgery and patient telemonitoring, which uses advanced data analytics and machine learning for decision-making and patient management. These applications aim to reduce surgical hours, increase accessibility to quality treatment, and enhance the safety and effectiveness of surgical education [29].

Similarly, the integration of IoT in blood glucose monitoring using a visible light source offers a non-invasive approach to diabetes management. This method uses a visible light sensor to detect glucose levels based on light interactions with the body, processing the data into glucose readings that are transmitted in real time via IoT connectivity to cloud-based platforms. Advanced algorithms analyze these trends to provide actionable insights, enabling real-time monitoring, alerts on abnormal glucose levels, and remote patient management. Such systems not only improve patient care by allowing continuous glucose monitoring without frequent clinic visits but also enhance patient compliance with educational content and medication reminders, making diabetes management more effective and less intrusive. Despite the rapid integration and evident benefits of IoT in both surgical practice and chronic disease management, the field faces challenges like data privacy, system security, and the need for further validation through comprehensive research. This systematic exploration within the paper aims to consolidate knowledge of IoST's current uses, identify gaps, and propose directions for future research.

Overall, the development of this non-invasive glucose monitoring device marks a significant advancement in diabetes management technology. By carefully selecting components that balance scientific effectiveness, cost, and user accessibility, the researchers have crafted a device that not only meets clinical needs but also enhances patient compliance and quality of life. This approach promises to transform how diabetes is managed daily, making it easier, and less intrusive for patients to monitor their glucose levels regularly.

## Conclusions

The blood glucose monitoring device's design has therefore been successfully tested and put into practice for non-invasive measurement. The detecting sensor measures the glucose levels of various patients. Laser light with a wavelength of 650 nm and a photodiode were used to produce it. By changing the laser's light intensity, the thickness of the patients is considered. The Arduino microcontroller controls its functioning. As a result, the glucose level is determined, and the resulting value (mg/dL) is presented. The results agree well with invasive Accu-Chek measurements as well as pathology lab data for nearly 2000 subjects of different age groups with diabetes and other complications.
